# Risk for *Mycobacterium celatum* Infection from Ferret

**DOI:** 10.3201/eid1703.100969

**Published:** 2011-03

**Authors:** Eva Ludwig, Udo Reischl, Thomas Holzmann, Holger Melzl, Dirk Janik, Constanze Gilch, Walter Hermanns

**Affiliations:** Author affiliations: Ludwig-Maximilians-Universität München, Munich, Germany (E. Ludwig, D. Janik, W. Hermanns);; Universitätsklinik Regensburg, Regensburg, Germany (U. Reischl, T. Holzmann, H. Melzl);; Tierärztliche Klinik Nürnberg Hafen, Nuremberg, Germany (C. Gilch)

**Keywords:** Disseminated granulomatous inflammation, ferret, molecular species typing, Mycobacterium celatum, 16S rDNA sequencing, Ziehl-Neelsen staining, tuberculosis and other mycobacteria, bacteria, letter

**To the Editor:** *Mycobacterium celatum* belongs to the group called “mycobacteria other than tuberculosis”; it is characterized by slow growth and a slender, rod-shaped form (0.25–0.5 × 0.5–13.0 μm). The cells are acid fast and do not form cords or branches. The species name, *celatum*, which means hidden or concealed, refers to the problem of phenotypically distinguishing the species from other mycobacteria, especially *M. xenopi. M. celatum* was first described in 1993 as a pathogen in persons with AIDS (*1*). Until now, few cases in humans have been reported; those cases were predominantly disseminated mycobacteriosis in immunocompromised patients (mainly those with AIDS), but they have also occurred in immunocompetent persons (*1**,**2*). For animals, 1 case of *M. celatum* infection in a ferret has been described (*3*). We describe another case in a ferret, with possible transmission to a human.

In 2009, a 3-year-old, neutered male, domestic ferret was examined in a veterinary clinic in Nuremberg, Germany, for a 5-month history of coughing, recent weight loss, reduced general condition, vomiting, and mild diarrhea. A chest radiograph showed multiple nodular densities in the lungs. Because of a poor prognosis, the ferret was euthanized. Necropsy was performed at the Institute of Veterinary Pathology in Munich. The lungs contained multifocal firm, light brown nodules, 6–10 mm in diameter (Figure, panel A). Spleen and lymph nodes (cervical, retropharyngeal, bronchial, gastric, mesenterial, popliteal) were enlarged. Histologic examination of lung, lymph nodes, spleen, liver, and brain showed granulomatous inflammation with predominantly macrophages, epithelioid cells (in the lung, including bronchioles), and some multinucleated giant cells. Several acid-fast bacilli were visible with Ziehl-Neelsen staining, mainly intracytoplasmically in epithelioid cells (including those of bronchioles) (Figure, panel B).

**Figure:**
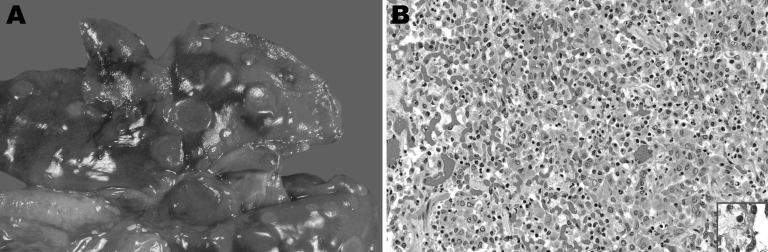
Appearance of tissue from 3-year-old, neutered male, domestic ferret with *Mycobacterium celatum* infection. A) Gross appearance: multiple, round light brown foci over lungs. B) Histologic appearance, granulomatous pneumonia: alveoli filled with foamy macrophages, epithelioid cells, and a multinucleated giant cell; also mild interstitial infiltration with lymphocytes, plasma cells, and neutrophils. Hematoxylin and eosin staining, original magnification x200. Inset, slender, rod-shaped, acid-fast bacilli in the cytoplasm of epithelioid cells; Ziehl-Neelsen staining, original magnification x400. A color version of this figure is available online (www.cdc.gov/EID/content/17/3/553-F.htm).

Conventional mycobacterial culture and PCR were used to look for mycobacteria in the lung, spleen, and lymph nodes. For culture, the material was homogenized, decontaminated, and spread onto solid Loewenstein-Jensen agar and injected into a liquid culture (Mycobacteria Growth Indicator Tube; Becton Dickinson, Heidelberg, Germany) for automated detection of mycobacterial growth.

DNA was extracted from the homogenized tissue by using the QiaAmp DNA Mini Kit (QIAGEN, Hilden, Germany), and a 510-bp fragment at the 5′ end of the ribosomal 16S rDNA was amplified as described (*4*). The amplified fragment of the expected length was sequenced, and data were analyzed by using the Integrated Database Network System (SmartGene Services, Lausanne, Switzerland; www.smartgene.com). The resulting sequence was clearly interpretable and unambiguously assigned to *M. celatum*; sequence identity to GenBank accession no. Z46664 was complete except for 1 mismatch in bp 490. Minor sequence diversity in the *M. celatum* 16S rDNA gene has been documented (*5*). The most closely related species, *M. kyorinense*, differs substantially, having 11 mismatches within the 16S rDNA gene (*6*). Species identity was further supported by phylogenetic analyses of the *hsp65* (*7*), *rpoB* (*8*), and *sodA* genes (*9*).

After 14 days of incubation, the solid and liquid cultures showed growth of acid-fast bacilli. Further identification at the Mycobacteria Reference Laboratory of the Bavarian Health and Food Safety Authority (Oberschleissheim, Germany) confirmed the molecular species typing results of *M. celatum*.

In Europe, naturally occurring mycobacterial infections in ferrets are rare; but in New Zealand, *M. bovis* or *M. avium complex* infections in ferrets are common (*10*). For ferrets, clinically relevant mycobacteria species are *M. genavense* and *M. microti*, among others. In humans, *M. celatum* mostly affects immunocompromised hosts with developing pneumonia or disseminated mycobacteriosis (*1*); inflammation in infected immunocompetent hosts is usually limited to the lungs or lymph nodes (*2*).

The ferret reported here had disseminated mycobacteriosis with no evidence of immunosuppression. In this respect, the clinical response of ferrets seems to differ from that in humans. The question of zoonotic risk remains. The ferret’s owner had had a cough for a long time, but radiography and sputum analysis showed no evidence of infection. However, these findings do not rule out mycobacteriosis because the owner had received unknown antimicrobial drug therapy before the samples for microbiology were collected. In general, potential transmission of mycobacteria should not be underestimated; in this case, intracytoplasmic bacilli were detected in the bronchioli of the ferret’s lung, making airborne spread of mycobacteria and infection of humans possible. The source of the primary infection in the ferret is not clear, but the dominant lesions in the lungs suggest it was airborne.

*M. celatum* infection must be strongly considered as a differential diagnosis in ferrets with pneumonia and generalized lymphatic hyperplasia. PCR and molecular species typing by 16S rDNA sequencing seem to be essential for an early and definitive diagnosis (*1*). The zoonotic risk for *M. celatum* infection in immunocompromised as well as immunocompetent persons should be kept in mind, considering the possible airborne transmission and the close contact between the animals and their owners.
